# Optimal Organelle Inheritance Strategies Under Different Changing Environments and Mutational Pressures

**DOI:** 10.1093/gbe/evag161

**Published:** 2026-06-27

**Authors:** Belén García-Pascual, Jan M Nordbotten, Iain G Johnston

**Affiliations:** Department of Mathematics, University of Bergen, Bergen, Norway; Department of Mathematics, University of Bergen, Bergen, Norway; Department of Mathematics, University of Bergen, Bergen, Norway; Computational Biology Unit, University of Bergen, Bergen, Norway

**Keywords:** mitochondria, plastids, organelle DNA, inheritance, mutation, changing environments

## Abstract

Mitochondrial and chloroplast DNA encode essential cellular apparatus. This organelle DNA exists at high copy number (ploidy) in eukaryotic cells, which must both mitigate mutational damage and allow adaptation to changing demands. Across eukaryotes, organelle DNA is inherited and maintained by different classes of processes. Inheritance is often maternal, but some species use paternal or doubly uniparental (sex-dependent) inheritance, with different extents of “leakage” of organelle DNA from the non-primary parent. During development, genetic bottlenecks of different magnitudes and recombination-mediated repair are employed in different species. Here, we use modeling and simulation to investigate the fitness advantages, disadvantages, conflicts, and tradeoffs of these different strategies under different challenges of mutation and changes in selection imposed by the environment (in the absence of interactions with nuclear genes). We find a general tradeoff between maintaining heteroplasmy to support adaptation to environmental change and supporting purifying selection against dysfunctional mutants. Different combinations of leakage and bottleneck size provide optimal resolutions to this tradeoff under different sets of challenges. We connect our findings to biologically observed behaviors, including the universality of non-minimal bottleneck sizes, a tradeoff between high ploidy for heteroplasmy and repair and tight bottlenecks for segregation, and environmental dependence of the benefits of leakage and doubly uniparental inheritance.

Significance Across eukaryotic life, different taxa have evolved a wide diversity of strategies for inheritance of organelle DNA genomes (oDNA; mitochondrial DNA and plastid DNA) between generations. Classic theoretical work has explored the behavior of these different strategies under constant or zero selective pressures from the organism's environment. However, the advantages and disadvantages of different inheritance and maintenance strategies under changing environments have remained comparatively underexplored—and are a central question both for basic genome biology and in the light of ongoing environmental change experienced by modern-day ecosystems. Here, we use a theoretical model coupling mutational hazard and different modes of environmental change (favoring different oDNA types) to explore the pros and cons of different strategies of mitochondrial and plastid inheritance. We identify strategies with the most power to preserve oDNA in different regimes of environmental change, and discuss this theory in the light of eukaryotic diversity and a changing biosphere.

## Introduction

Inheritance patterns of mitochondria and chloroplasts, and their internal organelle DNA (oDNA), are diverse across eukaryotes (Greiner et al. 2015; Munasinghe and Ågren 2023; Chung 2025). oDNA typically exists at high ploidy (hundreds or thousands of molecules per cell) and may be heteroplasmic, with an admixture of different oDNA variants within a cell (Wallace and Chalkia 2013; Johnston and Burgstaller 2019; Van den Ameele et al. 2020). How these multiple genomes are inherited between generations varies across lineages (Greiner et al. 2015; Munasinghe and Ågren 2023; Veeraragavan et al. 2024; Lane and Pomiankowski 2026). For example, some organisms follow the familiar human (and mammalian) pattern of strict maternal inheritance of mitochondrial DNA (mtDNA), with very limited or no “leakage” of mtDNA from the father, and little or no recombination. Some bivalves show doubly uniparental inheritance (DUI), where mothers contribute germline mtDNA to daughters and fathers to sons (Zouros et al. 1992, 1994; Passamonti and Ghiselli 2009). Inheritance of mtDNA and chloroplast DNA (cpDNA, also called ptDNA for plastid DNA) in plants and algae is often maternal but sometimes paternal (Fauré et al. 1994; Greiner et al. 2015; Munasinghe and Ågren 2023), and often involves leakage from the other parent (McCauley 2013; Breton and Stewart 2015). Plants show a dramatic amount of recombination in organelle DNA, involving templated repair and gene conversion processes (Khakhlova and Bock 2006; Wu et al. 2020; Broz et al. 2022, 2024; Zwonitzer et al. 2024). Fungi and protists also use oDNA recombination (Barr et al. 2005), and different lineages show different modes of inheritance, including biparental inheritance (Birky 2001; Greiner et al. 2015), leakage (Wilson and Xu 2012; Xu and Li 2015), and even inheritance of mtDNA independently of either nuclear parent (three-parent offspring; Bloomfield et al. 2019).

A particularly important aspect of organelle DNA inheritance is the “genetic bottleneck,” or the effective population size of organelle DNA molecules inherited by an offspring from its parent (Zhang et al. 2018; Johnston 2019a; Van den Ameele et al. 2020). As inheritance has a random component, smaller bottlenecks introduce more variability in the oDNA profiles of offspring; larger bottlenecks mean a closer resemblance between siblings (and parent). A genetic bottleneck, manifest through a physical reduction in mtDNA copy number and parallel stochastic processes, is used to segregate mutational damage between offspring in animals (Johnston 2019a). Plants also induce a genetic bottleneck, likely using a mechanism involving gene conversion and more limited physical reduction (Edwards et al. 2021; Broz et al. 2022, 2024). The inheritance of mtDNA in animals involves purifying selection (Fan et al. 2008; Stewart et al. 2008; Lieber et al. 2019; Palozzi et al. 2022; Kannangara et al. 2026), which is itself dependent on environmental cues (Latorre-Pellicer et al. 2019). Segregation of mutational load allows selection to act at the cellular or embryonic level, purifying population-wide oDNA populations (Krakauer and Mira 1999; Wallace and Chalkia 2013; Zhang et al. 2018; Johnston 2019a; Edwards et al. 2021). This purification helps remove dysfunctional mutant alleles, but also helps mitigate physiologically deleterious effects from cellular admixtures of oDNA (Lane 2012; Sharpley et al. 2012; Lechuga-Vieco et al. 2020) and potential longer-term disadvantages arising from conflicts between mixed symbionts (Frank 1996). MtDNA inheritance in animals, and presumably other taxa, thus involves an interplay of selection (within and between cells) and segregation (Burgstaller et al. 2018; Veeraragavan et al. 2024; Johnston 2026). One question here is: as tighter genetic bottlenecks segregate mutational damage more effectively, why do we rarely see genetic bottlenecks approaching a minimum size (one effective molecule inherited from generation to generation)?

Arguments have been put forward for the advantages and disadvantages of various inheritance strategies (Greiner et al. 2015; Munasinghe and Ågren 2023). Quantitative models have been studied for the action of recombination (Lonsdale et al. 1988; Atlan and Couvet 1993; Albert et al. 1996) and gene conversion (Walsh 1992; Edwards et al. 2021), and the bottleneck and mutation-selection balance (Bergstrom and Pritchard 1998; Roze et al. 2005). In addition to the above advantages of a genetic bottleneck and recombination, these arguments include uniparental modes limiting fitness costs associated with heteroplasmic populations (Hadjivasiliou et al. 2012; Lane 2012; Sharpley et al. 2012; Christie et al. 2015), redistributing mutational variance (Radzvilavicius 2021), and promoting adaptative evolution (Christie and Beekman 2017a, 2017b), and leakage helping circumvent “mother's curse” and preserving genetic variance in populations to mitigate against environmental change (Cosmides and Tooby 1981; Gemmell et al. 2004; Camus et al. 2012; Beekman et al. 2014; Connallon et al. 2018; Radzvilavicius et al. 2021). Theory has been advanced for how the evolution of an animal-like germline satisfies requirements for high-ploidy oDNA and mutational robustness (Radzvilavicius et al. 2016; Colnaghi et al. 2021), and how oDNA copy number shapes the relative influence of these different processes (Roze et al. 2005; Edwards et al. 2021). The maintenance of mitochondrial integrity in the face of different modes of damage is argued to be a defining reason for the evolution of sex (Lane and Pomiankowski 2026). Mito–nuclear interactions, where interplay between products of DNA from the nucleus and organelle influence organism phenotype (Wolff et al. 2014; Havird et al. 2019; Pozzi and Dowling 2022; Moran et al. 2024), are also predicted to shape the evolution of different inheritance modes (Hadjivasiliou et al. 2012, 2013; Radzvilavicius et al. 2017; Allison et al. 2021).

Many of these modeling approaches have considered either no or constant organismal-level selection. Here, we focus particularly on one class of proposed mechanisms supporting different modes of organelle DNA inheritance: adaptation to changing environments (Fraser and Kaern 2009; Tufto 2015). Different environmental conditions including temperature and altitude have been shown to favor different mtDNA variants in several species (Mishmar et al. 2003; Ruiz-Pesini et al. 2004; Luo et al. 2013; Consuegra et al. 2015; Le Cam et al. 2024, 2026; Graham et al. 2024). Emerging direct evidence from experimental evolution has demonstrated that changing temperature impacts mtDNA haplotype frequencies in populations (Lajbner et al. 2018), with implications for mitochondrial evolution in a changing world (reviewed recently in [Kannangara et al. 2026]). Previous work has argued that paternal leakage (Radzvilavicius and Johnston 2020) and bottlenecks (in addition to their function in purifying selection) (Radzvilavicius and Johnston 2022) can individually facilitate oDNA adaptation to changing environments; experimental work has shown that some environmental stressors influence plant organelle inheritance regimes (Chung et al. 2023; Gonzalez-Duran et al. 2026). However, to our knowledge, a synergistic treatment of these strategies together (along with others like DUI and recombination-mediated repair) and considering both mutational hazard and environmental change has yet to emerge.

Here, we attempt such a synergy through a stochastic modeling framework. We consider a portfolio of different organelle inheritance and maintenance strategies found across eukaryotes: bottlenecks, leakage, DUI, templated repair, and gene conversion. We ignore organelle–nuclear interactions, but consider joint mutational and environmental change challenges to populations as they evolve and transmit organelle DNA between generations. Our main question is, if an organism is faced with changing environments and a mutational burden, what combination of strategies is optimal for organelle DNA inheritance? We show that different balances and magnitudes of external challenges effectively favor different weightings of genetic priorities in the evolving population, and identify the different strategies that are optimal with respect to these priorities. We also connect with behavior observed across eukaryotic lineages and discuss the associated predictions that emerge from our model.

## Results

### Inheritance in Constant Environments

**Fig. 1.**
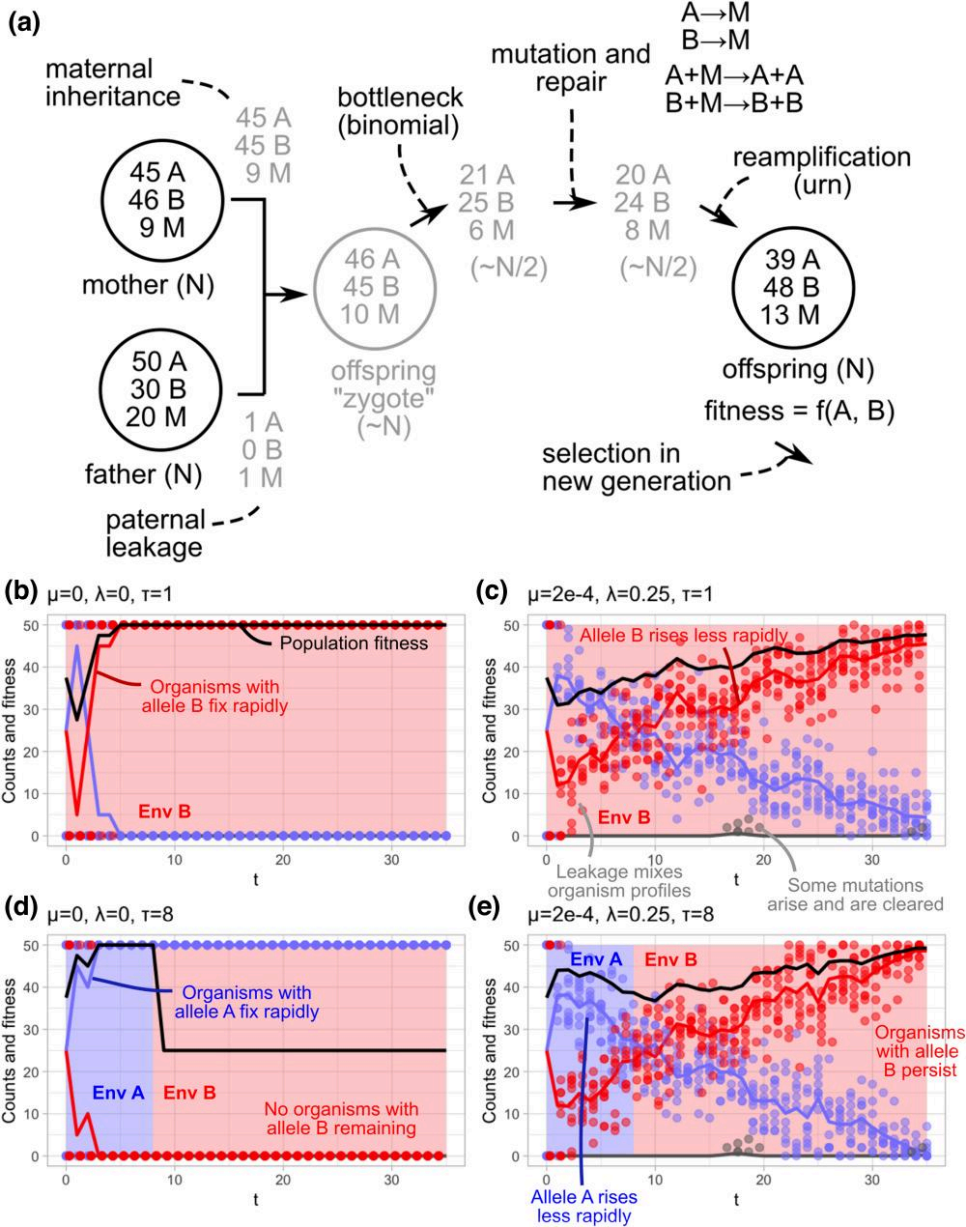
Model processes and illustrative behavior. a) *Constructing an offspring*. Parents, randomly selected based on fitness, have {A, B, M} oDNA profiles (brackets illustrate copy number at each stage, which may be exact (e.g. *N*) or approximate (e.g. ∼*N*, around *N* but with some sampling variability). These profiles are binomially sampled (potentially including leakage), combined, and subjected to a binomial bottleneck (Eqn. M1). Mutation (Eqns. M2 and M3) and repair (Eqns. M4 and M5) processes, if applicable, shape the offspring's oDNA profile. Reamplification via an urn-like model, possibly including within-cell selective differences between oDNA alleles (Eqn. M6), then produces an adult organism of the next generation. The process is repeated, sampling parents of the previous generation, until a new population of the same size is produced. Each is then assigned a fitness based on the fitness function and its oDNA profile, before selection for the next generation. b to e) *Example model behaviors in different conditions*. Points and traces give individual and population mean counts of A (blue), B (red), and M (gray) alleles. Other parameters (see Methods): population size *N*_pop_ = 100, oDNA population size *N* = 50, fitness from inferior allele *σ* = 0.5, heteroplasmy penalty *ε* = 0, selection coefficients *s*_B_ = *s*_M_ = 1, template repair rate *ρ* = 0. Black trace gives population mean fitness; background color shows allele with selective advantage. b) No mutation or leakage, constant environment: rapid fixation of the favored allele (red). c) Rare mutation and high leakage, constant environment: some mutational fluctuations and longer time to fix the favored allele (not fixed by the end of simulation). d) No mutation or leakage, environmental change in generation 8: rapid fixation of initially favored allele, no alternative allele retained, so later fitness drops. e) Rare mutation and high leakage with the same changing environment: leakage preserves heteroplasmy, allowing the system to adapt after environmental change.

As described in the Methods and illustrated in Fig. 1, we created a model of individuals evolving over discrete generations, where each individual can contain different copy numbers of organelle DNA types A, B, and M. Total oDNA copy number is *N* (for consistency with literature modeling mtDNA populations; we use *N*_pop_ for the number of organisms in a population). A and B are functional variants, and M is a dysfunctional mutant. An individual's fitness is determined by an environment-dependent fitness function, which has a unit contribution from either A or B (depending on environment), a lower contribution (parameter σ<1) from the other functional oDNA type, zero contribution from M, and a possible heteroplasmy penalty ϵ. The environment can change over time from favoring one functional type to the other—we will focus on the simple case of a single, irreversible environmental change, and later explore periodic changes. Offspring are produced by selecting two parents based on fitness, then binomial sampling of the mother's oDNA (either individually or in clusters of size $n_{c}$) with some paternal leakage (probability *λ* per oDNA), through a developmental bottleneck (size N/2). In the limiting case of λ=1/2, oDNA molecules are inherited with equal probability from the mother and father, making the sex labels redundant and capturing the case, for example, where two cells simply fuse and mix oDNA. The sampling of inherited oDNA may either be unbiased or biased by selection coefficients *s*_B_ and *s*_M_ for B and M (relative to 1 for A). Mutation, changing A and B to M, is applied randomly with a given rate parameter *μ* (broadly corresponding to damage rate per oDNA per generation), and templated repair, overwriting one oDNA type with a template from another, is applied randomly with a rate parameter *ρ*.

**Fig. 2.**
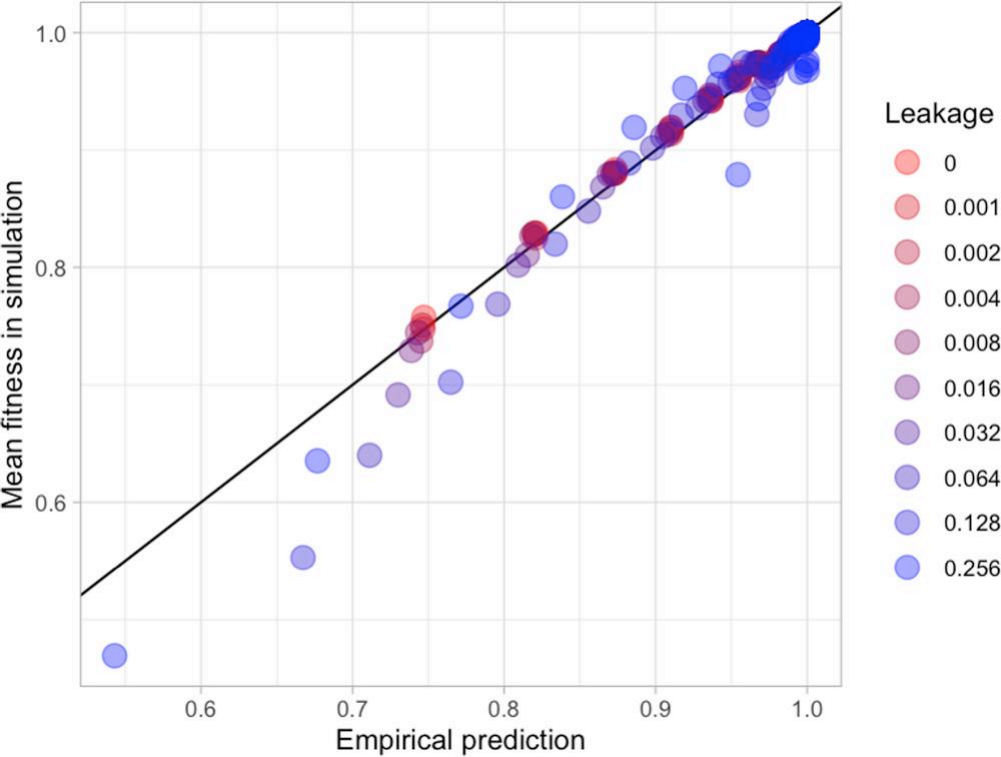
Empirical fit to constant environment behavior. The horizontal axis gives an empirical prediction for mean fitness from the argument in the text; the vertical axis gives fitness observed in simulations. Colors give different leakage rates; points within a color correspond to different *μ* and *N* values for that leakage. *R*^2^ = 0.96 for the predicted-observed link, but higher leakage values are less well correlated with prediction. Other parameters *N*_pop_ = 100, *σ* = 0, *τ* = 1, *ε* = 0, *s*_B_ = *s*_M_ = 1, *ρ* = 0.

We first examined the “baseline” mutation-selection behavior of our model in the absence of changing environments. Previous work has derived expressions related to equilibrium fixation probabilities in a similar model (Roze et al. 2005), but to characterize our numerical model, which does not necessarily reach an equilibrium state within the timeframe of simulation, we took a more empirical approach. We simulated the situation where *σ* = 0, *ε* = 0, and *τ* = 1 in Eqn. M9 (allele B has unit fitness and A and M have zero fitness). Example trajectories are shown in Fig. 1b and c. Across different values of paternal leakage and oDNA ploidy (which corresponds with bottleneck size), we found that the general expression


(1) $$f\approx 1-\;\frac{\mu \;\sqrt{N}}{{\left(1\;-\;\lambda \right)}^{2}}$$

well described long-term mean fitness *f* (Fig. 2; *R*^2^ = 0.96 across different organism population sizes, although larger leakage rates are less well correlated with the prediction).

We do not aim to derive Eqn. 1 from first principles, but its form admits some intuitive analysis. First, in the case of no leakage and a single oDNA per organism, an organism's fitness is determined entirely by the single allele it possesses, and we retrieve the expected result from classical population genetics that f=1−μ. The dependence on oDNA copy number *N*—higher populations had marginally lower mean fitness for a given mutation rate—is a consequence of a weaker “bottleneck” acting to generate selectable variability in mutant load between generations. In the infinite-*N* limit each offspring clonally inherits its mother's mutant load; the variance of inherited mutant load *h* under binomial inheritance follows Var(*h*) ∼ 1/*N*, suggesting an interpretation of the $\sqrt{N}$ term—the associated standard deviation—as a measure of the selectable “spread” of values.

Nonzero leakage allows the bottleneck to be circumvented, supporting the maintenance of some mutant alleles that would otherwise be more efficiently purged by selection. The (1−λ) empirical term in this sense resembles the (1−μ) term above and in classical population genetics.

### Inheritance in Changing Environments

**Fig. 3.**
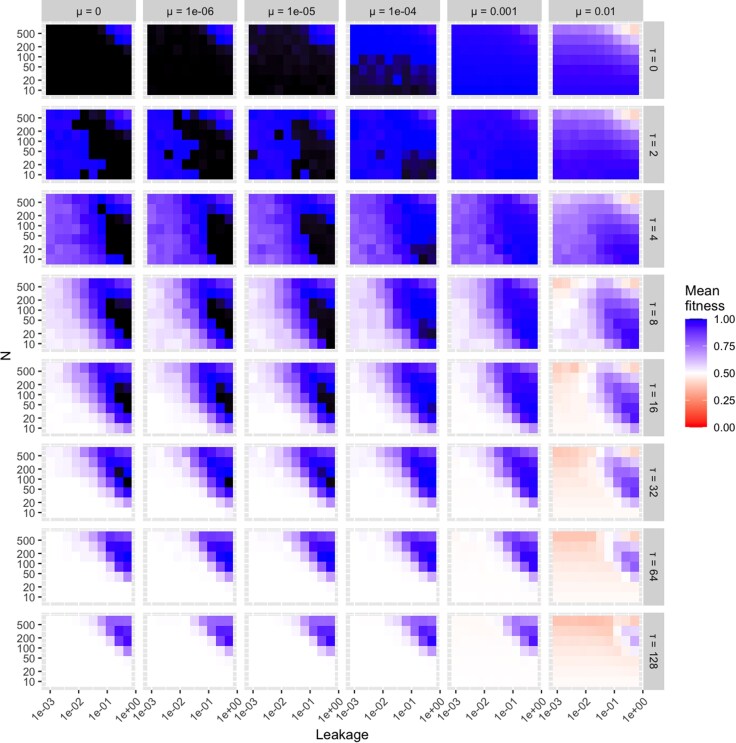
Fitness with different inheritance strategies under different challenges. Columns of the trellis give mutation rate *μ*, rows give time to environmental change event *τ*. In each panel, the horizontal axis is leakage rate and the vertical axis is oDNA copy number. Fitness is given by color: 0.5 (white) corresponds to inferior allele performance, lower values correspond to contributions from dysfunctional mutants. Maximal fitness of exactly 1 is given by black pixels. Templated repair and DUI are both absent and there is no heteroplasmy penalty; organismal population size is 100 (parameters *N*_pop_ = 100, *σ* = 0.5, *ε* = 0, *s*_B_ = *s*_M_ = 1, *ρ* = 0). Fig. S2 shows comparable plots for alternative cases.

Having established this baseline behavior, we turned to our main research question: which inheritance strategies are optimal under different regimes of environmental change? To this end, we simulated the population with a time-varying fitness function which changes once and irreversibly from favoring allele A to favoring allele B after a given time window (example trajectories in Fig. 1d and e). We explored how the subsequent fitness of the population behaved under different time windows for environmental change and different mutation rates, as a function of paternal leakage rate and oDNA population size (Fig. 3).

The case of zero environmental change (top row of Fig. 3) maps identically to the results in the previous section (Eqn. 1). For zero mutation rate, fitness is maximal in all cases except high *N* and high leakage, which increases the timescale necessary for complete adaptation beyond the scale of the simulation. Low *N* and low leakage reduce this timescale, leading to rapid fixation of the environment-matched allele. Low but nonzero mutation rates can be absorbed by the system, with the bottleneck acting to segregate mutants, which can then be purged by cell-level selection. This negative selection is less effective for higher *N* (looser bottleneck), where the mutations that are not purged shift the system from perfect adaptation. At higher mutation rates (>10^−4^), even tight bottlenecks do not allow full purging of mutation, and some mutational entropy is always present in the system.

Any degree of environmental change changes this profile (lower rows of Fig. 3). Now, too-rapid fixation of the allele matched to the first environment becomes negative, as it removes from the gene pool the allele that is matched to the later environment. Hence, low *N*, low leakage behavior is less optimal than higher leakage, which preserves heteroplasmy for longer in the population and hence allows later fixation of the allele matching the second environment. Lower *N* values support the more rapid fixation of this second allele, with higher *N* values shifting from optimality as this fixation happens more slowly. For nonzero mutation, higher *N* values are further disfavored as they diminish the power of the bottleneck to segregate mutation for subsequent purging. Low *N*, high leakage strategies are therefore favored.

As the period of environmental change gets longer, the lowest *N* values become disfavored, as they lead to too-rapid fixation of the first allele and loss of heteroplasmy before the environmental change. There are then competing pressures on *N* even for zero mutation: too low and we lose heteroplasmy, too high and we fail to adapt quickly to the later environment. For nonzero mutation, as before, high *N* values are further disfavored due to a weak bottleneck's inability to segregate damage. The priority of preserving heteroplasmy leads to a coupling between leakage rate and *N*: high *N*/low leakage and low *N*/high leakage strategies allow the same heteroplasmy, but high *N*/high leakage is challenged by the slow rate of later adaptation. As low *N* is valuable for purging mutations, we see low *N*/high leakage emerge as the optimal strategy under these conditions, with high *N*/low leakage a less optimal alternative, and other combinations substantially worse.

**Fig. 4.**
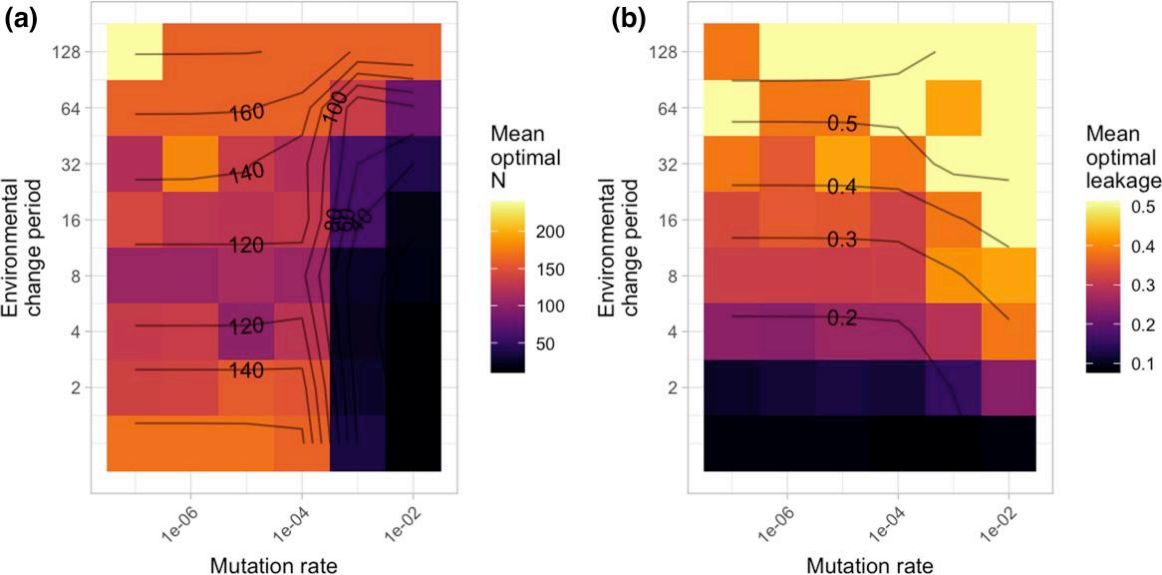
Optimal inheritance strategies under different challenges. a) Mean *N* value across strategies giving maximal fitness for a given mutation rate and environmental change frequency (zero on bottom row of pixels). An overall range of *N* from 1 to 512 was sampled. b) Mean leakage rate across strategies giving maximal fitness. Contours are interpolated from a 2D LOESS fit across sampled values. Simulation parameters *N*_pop_ = 100, *σ* = 0.5, *ε* = 0, *s*_B_ = *s*_M_ = 1, *ρ* = 0.

These trends are summarized in Fig. 4, where the optimal strategies for a given mutation rate and environmental change period are summarized. Optimal *N* decreases monotonically with mutation rate, reflecting the importance of the bottleneck for subsequent purging of mutation. For lower mutation rate, optimal *N* shows re-entrant behavior with environmental change period: shorter and longer changes support higher *N*, while intermediate changes support lower *N*. This re-entrant observation is a result of averaging: the range of optimal *N* values is wider for shorter environmental change periods, and the average over all *N* that give maximal fitness is reported here. Optimal *N* becomes more constrained at low values for intermediate periods, and equally constrained to higher values at longer periods, so the trend is “flexible-lower-higher.”

The behavior of optimal leakage is more straightforward, increasing strongly and monotonically during the environmental change period (supporting the maintenance of heteroplasmy), and weakly and monotonically with mutation rate. This latter effect again comes from averaging: at low mutation rates, there is a wider range of leakage values that support optimal behavior (hence a lower average value), while this gets more constrained to higher values as mutation rate increases.

### Influence of Different Population Structures and Inheritance Regimes

**Fig. 5.**
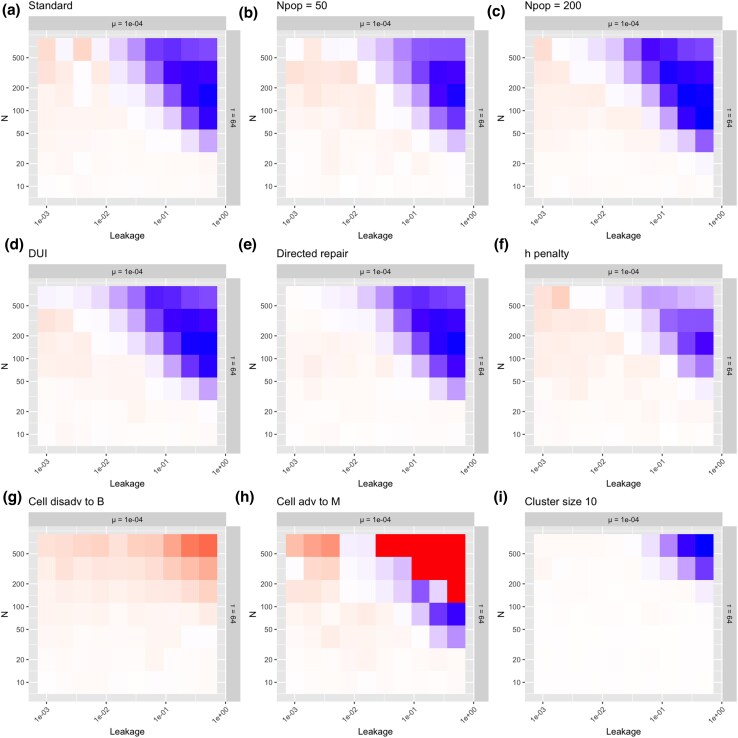
Influence of model variations. To demonstrate the influence of these effects, we focus on a particular instance of external challenge from Fig. 3 (environmental change period 64, mutation rate 10^−4^), though the trends we see here are reflected in other results (Fig. S2), including in their clearest form at a high model mutation rate (Fig. S3). Mean fitness is plotted as in Fig. 3, with the same color scale (blue, high fitness; red, low fitness) except the shades of red used have been darkened to emphasize deleterious regions. a) Default parameterization (*N*_pop_ = 100, *σ* = 0.5, *ε* = 0, *s*_B_ = *s*_M_ = 1, *ρ* = 0, as in Fig. 3). b) Smaller (*N*_pop_ = 50) and c) larger (*N*_pop_ = 200) organismal population. d) Doubly uniparental inheritance. e) Templated repair with rate constant *ρ* = 10^−3^. f) Heteroplasmy penalty *ε* = 0.25. g) Within-cell selective disadvantage to B (*s*_B_ = 0.9). h) Within-cell selective advantage to M (*s*_M_ = 1.1). i) Inheritance of clusters of size *n*_c_ = 10 rather than individual molecules.

The analysis above is a basic, foundational model with fixed organismal population size, uniparental inheritance, no explicit heteroplasmy penalty for individuals, and no templated repair of mutational damage. We next asked how the profile of optimal strategies changed when we relaxed these assumptions (Fig. 5).

The influence of organismal population size and DUI is dominated by the strength of maintaining heteroplasmy in a population. A smaller (0.5×) number of organisms has reduced capacity to retain heteroplasmy and exhibits generally lower fitness (Fig. 5b), with high fitness constrained to a small region of strategy space and optimal behavior slightly shifted to higher *N* (preserving heteroplasmy taking priority over the bottleneck). Conversely, a larger (2×) number of organisms has an increased capacity to retain heteroplasmy and performs better across a wider range of strategies (Fig. 5c), with optimal *N* shifted slightly downwards. Populations supporting DUI show only some subtle advantages resembling the trend from the larger population size (Fig. 5d; Fig. S2d). Slight improvements can be seen at high leakage, which couples the otherwise separated sex-specific “pools” from which oDNAs can be inherited and may allow a marginally larger effective source population of oDNA.

The introduction of directed templated repair (Fig. 5e) removes (to an extent dependent on parameterization) the need for a bottleneck. This is because mutations can now be removed at the within-organism (intracellular) level, rather than relying on segregation across offspring followed by organism-level selection. As such, the pressure against high *N* due to weaker bottlenecking is largely removed. High *N* still challenges rapid fixation of the final allele, but this effect is offset both by the increased stoichiometric availability of wildtype oDNA to template repair of mutants (Zwonitzer et al. 2024) and by the increased capacity to preserve heteroplasmy. With some amount of templated repair, we thus see a substantial shift toward higher optimal *N*, and diminished re-entrant behavior in *N* for a given leakage rate. At other combinations of environmental change and mutation rate (Fig. S2f), re-entrant behavior of fitness with oDNA ploidy is observed for higher mutation rates. High ploidy allows many templates for repair and low ploidy allows a strict bottleneck for segregation, but intermediate ploidy presents neither advantage as strongly and thus has a marginally lower fitness—an effect that is amplified with within-cell competition (below). Undirected repair, where mutants can overwrite wildtype alleles as well as vice versa, had little overall effect on fitness profiles (Fig. S2g).

Introducing an explicit fitness penalty for heteroplasmy provides a near-universal challenge to the system, and fitnesses are generally lower and optimal behavior much more constrained (Fig. 5f). The maintenance of heteroplasmy now comes at a fitness cost, promoting rapid fixation of the early allele and challenging the ability of the population to maintain and adapt the later allele. However, a small (depending on parameterization) region of strategy space involving high leakage and intermediate ploidy (and hence intermediate bottleneck size) is capable of retaining some fitness even in the challenging case demonstrated across Fig. 5. Here, the relative evolutionary advantage of heteroplasmy for later adaptation balances the fitness cost to individuals of a heteroplasmic admixture. Evolution cannot of course “look ahead” to anticipate future change, but this result shows that a population adapted to leakage will not necessarily go extinct in this scenario when faced with an mtDNA admixture giving a strong heteroplasmy penalty (see Discussion).

The presence of within-cell selection (Eqn. M6) couples with population-level selection to give several emergent structures of mean fitness. Figure 5g and h demonstrates some first effects, with more detail and the specific dependences on different environmental periods and mutations rates more completely visualized in Fig. S2. Several intuitive outcomes are observed: a within-cell disadvantage to the allele favored in the long-term environment (Fig. 5g, Fig. S2h) generally decreases long-term fitness as this allele is removed from the population (particularly with high leakage and ploidy); a within-cell advantage to this allele correspondingly improves long-term fitness (Fig. S2i). A within-cell disadvantage to the dysfunctional mutant allele (Fig. S2j) increases long-term fitness, particularly at high mutation rate and high ploidy; this influence is negligible at low ploidy, when a tight bottleneck can segregate mutant content sufficiently for population-level selection to act. A within-cell advantage to the dysfunctional mutant (a “selfish mutant,” Fig. 5h, Fig. S2k) dramatically amplifies the influence of ploidy on fitness: high ploidy now allows the selfish mutant to proliferate and leads to a dramatic fitness drop, while low ploidy enforces a strict bottleneck and allows between-cell selection to compensate for the within-cell advantage. The inheritance of clusters of molecules, rather than individuals (hence, clusters of size $n_{c}$ rather than individuals in Eqn. M1) acts effectively like a tighter bottleneck (Fig. 5i, Fig. S2l). Indeed, theory shows that segregation scales like the size of inherited clusters (Cao et al. 2007; Edwards et al. 2021), shifting the system's behavior to what would be observed for smaller copy numbers. In the case of cpDNA, which is typically highly polyploid but genetically homogeneous within organelles (Johnston 2019b), this mechanism supports the dramatic scales of segregation observed in plants (Broz et al. 2022, 2024) despite the large cellular copy numbers involved.

Overall, the effects of different directions of multilevel selection then provide conflicting pressures shaping the population (Gitschlag et al. 2020, 2024; Røyrvik and Johnston 2020; Yu et al. 2020; Kotrys et al. 2024), with the capacity for between-cell selection dependent on bottleneck size. The magnitude of leakage contributes to the potential for genomic conflict here. With a selfish mutant, high paternal leakage amplifies the fitness detriment, helping the mutant allele persist and proliferate in the population. The worst case is high ploidy (weak bottleneck) and high leakage with a selfish mutant, corresponding to little capacity for between-cell selection to combat the within-cell advantage.

### Emergence of Different Strategies in Evolutionary Simulation

The exploration of which inheritance strategies ([*N*, *λ*] pairs in our model) are optimal in the sense of maximizing mean fitness after an environmental change does not immediately describe how likely such strategies are to appear in an evolutionary setting. To explore this question, we next ran evolutionary simulations where organisms with different strategies compete while exposed to a common, dynamic environment. The structure of simulation is as before, except an offspring now also inherits *N* and *λ* from the parent from which it inherits the majority of its organelles. In one set of evolutionary experiments, we impose the same single-switching environmental protocol as before (Eqn. M9). In another set of experiments, we impose periodically oscillating environments, where the environment repeatedly switches between favoring allele *A* and favoring allele *B*, with period *τ* (Eqn. M10).

**Fig. 6.**
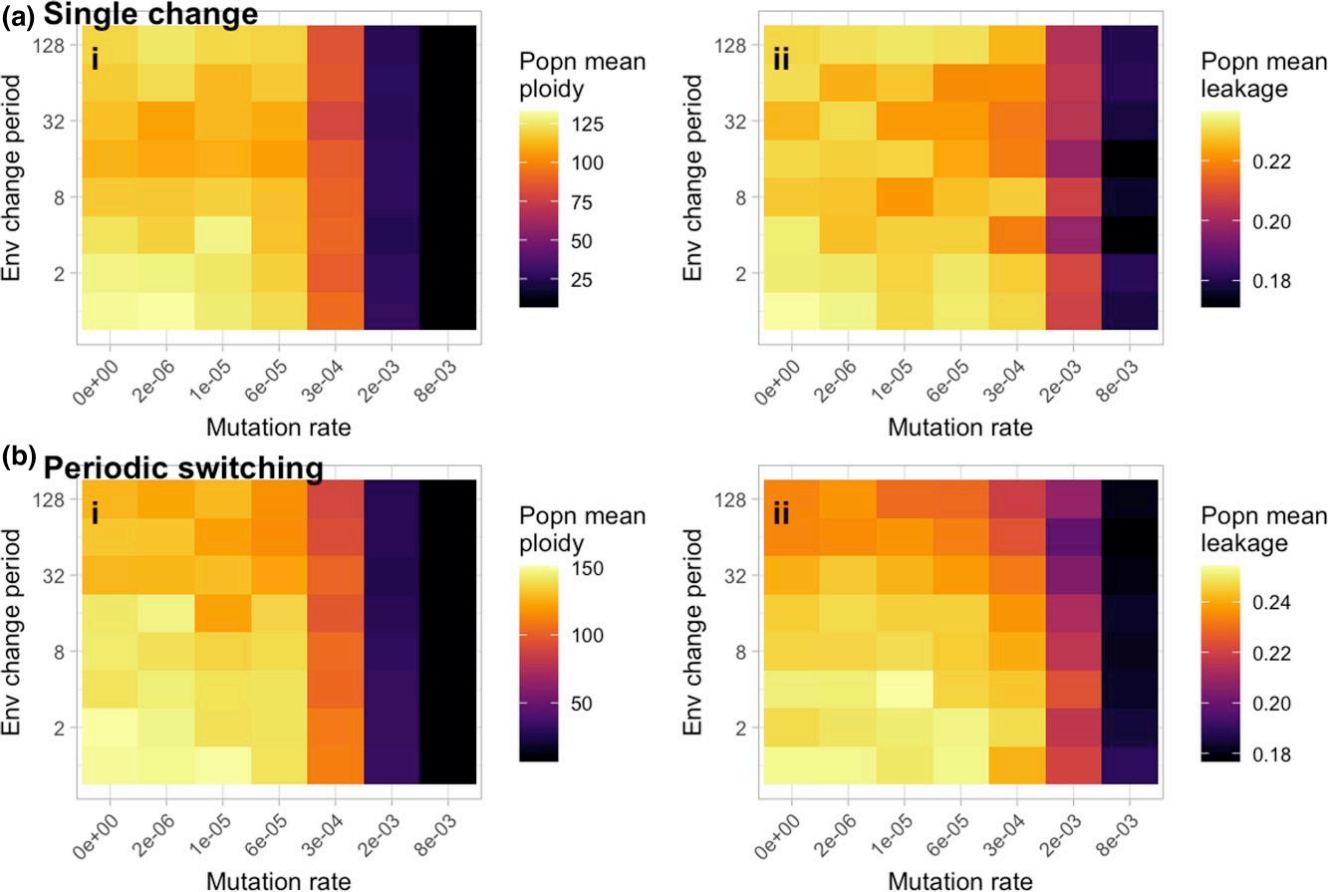
Emerging strategies in evolutionary simulation. Mean oDNA ploidy and leakage rate across populations after 300 generations under different environmental and mutational challenges. Parameters *N*_pop_ = 1,000, *σ* = 0.5, *ε* = 0, *s*_B_ = *s*_M_ = 1, *ρ* = 0. a) Mean ploidy *N* (i) and leakage *λ* (ii) for simulations with a single environmental change after one period, then a constant environment, matching previous experiments in the text. b) Mean ploidy *N* (i) and leakage *λ* (ii) for simulations with repeated environmental switching with the given period.

Figure 6 shows the mean (*N*, *λ*) across the population after 300 generations, averaged over 10^3^ independent simulations for each environmental period and mutation rate. The results for *N* with a single environmental switch (Fig. 6a) reflect the predictions from the optimal fitness simulations (Fig. 4a), with smaller *N* (tighter bottlenecks) strongly favored as mutation rates increase, higher *N* at large and small environment change periods, and lower *N* at intermediate change periods. The results in the repeated environmental switching simulation (Fig. 6c) are similar but without the favoring of higher *N* at longer environmental periods and low mutation rates. The repeated environmental challenges to the population here may make it harder for any strategy to retain sufficient genetic diversity for continued adaptation, and this trend may then reflect an “average” behavior with no particularly advantageous strategy.

Under these simulated conditions, the results for paternal leakage *λ* do not directly correspond to our identification of the optimal strategy from mean fitness calculations (Fig. 4b). The leakage rate emerging from simulation shows little diversity overall (typically between 0.18 and 0.25), while the predicted optimal rates vary with environmental change and mutation rate from 0.1 to 0.5. This suggests that sufficient pressure may not exist in the evolutionary system to adapt to the predicted optimal *λ* strategy. However, the small trend that does exist (a decrease in *λ* at higher mutation rates) is in disagreement with the predicted direction of the trend from Fig. 4b, where marginally higher *λ* values are optimal at higher mutation rates. In these circumstances, the predicted long-term optimal leakage strategy may therefore not be robust to shorter-term invasion by organisms with lower *λ* values.

## Discussion

**Fig. 7.**
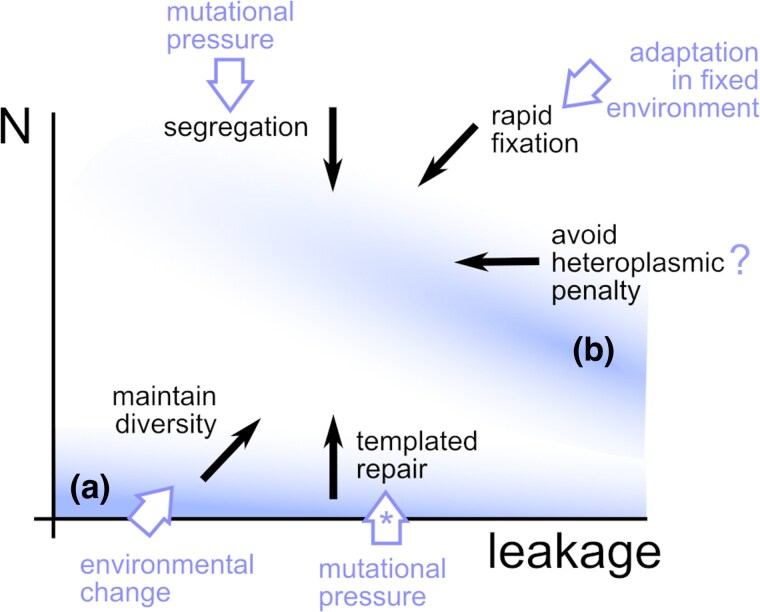
Summary of influences and strategies in our oDNA inheritance model. Thin arrows give the directions of genetic priorities for a given system; the relative weightings of these effects determine the optimal behavior. Thick arrows illustrate how different external pressures increase the relative importance of some priorities. Shaded regions illustrate a) optimal region for static environments (Eqn. 1); b) optimal region for changing environments. (?) The relative importance of avoiding heteroplasmy is hard to quantify on the same scale as the other values involved here, so this trend is presented semi-quantitatively. (*) Templated repair is not supported by some lineages, in which case mutational pressure will typically favor lower *N* for bottleneck segregation and clearance.

The maintenance of organelle DNA in the face of environmental change and mutation rate involves several challenges with solutions that are sometimes competing. To preserve heteroplasmy for adaptation to future challenges, rapid fixation of a single allele is not desirable—but rapid adaptation to current conditions is clearly positive. Preserving heteroplasmy within individuals is easier with larger organelle population sizes—but this decreases the power of the bottleneck to segregate mutational damage for clearance. There may be a conflict between levels of selection on short and long time scales: the rapid adaptation of one allele due to organismal fitness in the present may reduce the capacity of the population to survive future environmental change. There may also be a conflict between levels of selection on small and larger physical scales: an allele may experience a within-cell replicative advantage but be detrimental at the organismal level. The relative importance and influence of these challenges depend on their parameterization (biologically, the specific balances of rates, timescales, and other quantities), which varies tremendously across taxa and environments. But the direction of several of the challenges and strategies we study here can be summarized in Fig. 7.

What predictions can we extract from the model for biological interrogation? In agreement with previous work (Radzvilavicius and Johnston 2020), lineages subject to more dramatic environmental change would be predicted to favor the preservation of heteroplasmy through paternal leakage and DUI. Qualitatively, this agrees with, for example, the common observation of leakage in plants (Greiner et al. 2015) and DUI in bivalves (Passamonti and Ghiselli 2009), both of which are sessile in dramatically changing environments. Environmental stress—specifically chilling stress—upregulates paternal leakage in plants (Chung et al. 2023). Heteroplasmy has long been observed in these lineages (Pearl et al. 2009; McCauley 2013), as well as algae (Coyer et al. 2004) and fungi (Barr et al. 2005) often with similarly sessile lifestyles. In the limit of high leakage, oDNA inheritance is biparental—simple mixing of the oDNA content of two parental cells. Motile animals seem in general to exhibit fewer heteroplasmy-inducing and recombination strategies—although, for example, some degree of leakage, heteroplasmy, and recently recombination-mediated repair has been observed in Drosophila (Nunes et al. 2013; Klucnika et al. 2023). Excitingly, recent advances in synthetically controlling inheritance strategies in plants (Chung et al. 2023) will afford the opportunity to test these hypotheses more directly.

What determines the optimal oDNA copy number and bottleneck size in a given species? As illustrated in Fig. 7, several influences compete. Low *N* is beneficial for an animal-like bottleneck, although a bottleneck due to unbiased gene conversion can segregate damage independent of *N* (Walsh 1992; Johnston et al. 2015; Edwards et al. 2021). High *N* is beneficial for templated repair (effectively, biased gene conversion): when mutant oDNAs are preferentially overwritten by wildtype oDNAs, having stoichiometrically more wildtype oDNAs is beneficial for mutant loads under 50% (Zwonitzer et al. 2024). High *N* is also beneficial for maintaining heteroplasmy, as shown throughout this model. The particular *N* in a lineage then controls the balance between segregation due to an animal-like physical bottleneck (lower *N*) and repair of mutational damage (higher *N*) in that lineage, along with maintenance of heteroplasmy (higher *N*), with gene conversion supporting segregation (independent of *N*) (Edwards et al. 2021; Broz et al. 2022, 2024). Mechanisms like the inheritance of clusters of oDNA (Cao et al. 2007; Edwards et al. 2021) partially “decouple” copy number and variance generation. One notable example is plastid inheritance in plants: plastids are large, and a low number are inherited between generations. Although a plastid contains many ptDNAs, these appear quite genetically homogeneous (likely due in part to recombination), so the effective bottleneck size for plastid inheritance can be as low as 1 (Broz et al. 2022, 2024). By contrast, mitochondria are smaller and more genetically distinct (in plants, mitochondria contain on average less than one mtDNA; Preuten et al. 2010), with the effective bottleneck size correspondingly larger (Broz et al. 2022, 2024). Examples from plants indeed show tremendous variety in mtDNA copy number across species (Zwonitzer et al. 2024), with both repair and segregation facilitated by recombination machinery (Wu et al. 2020; Broz et al. 2022, 2024). The balance of priorities between mutation rate, environmental change, and the efficiencies of intracellular repair versus cell-to-cell segregation of damage will then determine the optimal *N* in a given setting. In this sense, our model mirrors (on a much smaller scale) the picture developed in Lane and Pomiankowski (2026), where adaptation to deal with different modes of mtDNA damage drives the evolution of the sexes themselves.

We have focused on genetic behavior in our model, where a more fine-grained perspective would consider more aspects of physical behavior both of organelles and of organisms. The physical dynamics of organelles influence both segregation and inheritance of oDNA (Tam et al. 2013, 2015; Aryaman et al. 2019; Edwards et al. 2021; Insalata et al. 2022; Glastad and Johnston 2023; Chung 2025), and the capacity for gene conversion and templated repair in recombining systems (Chustecki et al. 2021, 2022; Edwards et al. 2021; Giannakis et al. 2022). We the exception of inheritance of clusters of oDNA (likely reflective of chloroplasts), our coarse-grained model for the ongoing genetic bottleneck ignores the physical and genetic details of how corresponding oDNA variability is generated, preventing a direct connection to the rates of the processes involved (Birky 2001; Johnston and Jones 2016). Our model also lacks a distinction between germline and somatic tissue, thereby neglecting contributions to organismal fitness from somatic oDNA populations. This is of particular importance in the case of DUI, where—for example in mussels—somatic tissue may contain one parental mtDNA type while the germline (which we effectively model) contains the other (Breton et al. 2007, 2022; Passamonti and Ghiselli 2009). Expansion of this model to include germline–soma distinctions for multicellular eukaryotes, and the microscopic mechanisms of repair, segregation, and inheritance will be necessary to connect more quantitatively with true rates of these processes (Edwards et al. 2021). Another future target would be to explore different modes of environmental change. We have focused on a single shift and periodic shifts (in the evolutionary simulation); behavior under randomly varying timescales and magnitudes of environmental challenge would be a natural, though mathematically more complex, extension of this picture.

Interactions between organelle and nuclear genes are also absent from our model, which considers only the behavior of the organelle DNA population. The importance of mito–nuclear interactions are increasingly being revealed (Wolff et al. 2014; Havird et al. 2019; Pozzi and Dowling 2022; Moran et al. 2024) and indeed theory has shown that mito–nuclear interactions ay shape the evolutionary appearance of different inheritance strategies (including paternal leakage) and heteroplasmy (Hadjivasiliou et al. 2012, 2013; Radzvilavicius et al. 2017; Allison et al. 2021). However, the focus of this study was a general look at the many degrees of freedom associated with the organelle population alone; interactions with nuclear content and sex—which will provide additional contributions to these predicted dynamics, but will not necessarily invalidate them—will form a natural extension of this work. More generally, the collection of different influences in this system will reward follow-up study in combination. Combinations of templated repair and selfish mutant proliferation, for example, will be interesting to explore in more quantitative detail, especially in the framework of evolutionary simulation where strategies are allowed to compete with each other through evolutionary history.

## Methods

### Model Dynamics

The system consists of a population of *N*_pop_ organisms evolving over discrete generations (Fig. 1a). *N*_pop_ = 100 by default. An organism is described by three values {A,B,M} describing the copy number of three alleles in its organelle DNA population: *A* and *B* (functional variants, which may have different fitness contributions in different environments), and *M* (dysfunctional variant). Organismal oDNA copy number N=A+B+M. An organism is also assigned a sex, male or female, chosen so that exactly half the individuals in a generation are male and half are female. An organism's fitness is given by a fitness function f(A,B) described below, with allele *M* never contributing to fitness. Time proceeds through discrete generations. A new organism, in the absence of DUI (see below), is created as follows. A female mother and a male father are selected to become parents with probabilities proportional to their fitness values. Prior to mutation, offspring oDNA molecules are inherited with probability λ/2 from the father and with probability (1−λ)/2 from the mother to give offspring profile $\left\{A{\;}^{\prime },B{\;}^{\prime },M{\;}^{\prime }\right\}$:


[M1] $$\begin{array}{rl} & A^{\prime }\;∼\;\mathrm{Bin}(A_{F},\lambda /2)\;+\mathrm{Bin}(A_{M},(1-\lambda )/2)\\ & B^{\prime }\;∼\;\mathrm{Bin}(B_{F},\lambda /2)\;+\mathrm{Bin}(B_{M},(1-\lambda )/2)\\ & M^{\prime }∼\;\mathrm{Bin}(M_{F},\lambda /2)+\mathrm{Bin}(M_{M},(1-\lambda )/2) \end{array}$$

where Bin(*n*, *p*) is the binomial distribution, drawing individuals from a population of size *n* with probability *p*. Inheritance may also take place using clusters of size $n_{c}$ rather than individuals molecules, which are binomially partitioned as in Eqn. M1. Asymmetric mutations, causing A→M and B→M, are then applied with rate *μ* per molecule, causing copy number changes:


[M2] $$\begin{array}{rl} & \Delta_{\mu }A^{\prime }∼-\mathrm{Bin}(A^{\prime },\mu )\\ & \Delta_{\mu }B^{\prime }∼-\mathrm{Bin}(B^{\prime },\mu )\\ & \Delta_{\mu }M{\;}^{\prime }=\;-\Delta_{\mu }A^{\prime }-\Delta_{\mu }B^{\prime } \end{array}$$

leading to temporary oDNA profile $\{A{\;}^{\prime }+\Delta_{\mu }A{\;}^{\prime },B^{\prime }+\Delta_{\mu }B^{\prime },M^{\prime }+\Delta_{\mu }M^{\prime }\}.$

To simulate intracellular templated repair with rate *ρ* (ρ=0 by default), we choose a number of molecules in an organism to be randomly “overwritten” by randomly chosen template molecules. This models the processes X+Y→X+X, X+Y→Y+Y. We model two possibilities: (i) undirected, where any molecule (including mutants) can overwrite any other; and (ii) directed, supporting only overwriting *M* by *A* and *B*. In the undirected case, repair will be more likely if stoichiometrically more *A* and *B* are available (Fig. 1a, Zwonitzer et al. 2024). A number of overwriting events is chosen, given $N{\;}^{\prime }$ possible target molecules:


[M3] $$n_{\rho }∼\text{Bin}(N{\;}^{\prime },\rho )$$

For each event, one molecule is chosen randomly from the source population, and one from the target population. The first is duplicated and the second is removed, modeling overwriting. The changes in copy numbers from the $n_{\rho }$ instances of this process are written


[M4] $$\left\{\Delta_{\rho }A{\;}^{\prime },\Delta_{\rho }B{\;}^{\prime },\Delta_{\rho }M{\;}^{\prime }\right\}$$

So that the updated cell state is then:


[M5] $$\left\{A,B,C\}\to \{A{\;}^{\prime }+\Delta_{\mu }A{\;}^{\prime }+\Delta_{\rho }A{\;}^{\prime },B^{\prime }+\Delta_{\mu }B{\;}^{\prime }+\Delta_{\rho }B{\;}^{\prime },M^{\prime }+\Delta_{\mu }M{\;}^{\prime }+\Delta_{\rho }M{\;}^{\prime }\right\}$$

Finally, random “relaxed replication” of individual oDNA molecules re-amplifies the oDNA population (Chinnery and Samuels 1999). At each step of this process, an oDNA molecule is randomly chosen from the population and replicated. These steps are repeated until the total oDNA copy number reaches *N*.

The molecule chosen for replication depends on whether within-cell selective differences are present between allele types. If not, a molecule is randomly drawn from the current cellular population. If selective differences are present, the probability that a molecule of a given allele type is drawn is


[M6] $$\begin{array}{rl} & P(A)\;=\;A/(A+s_{B}B+s_{M}M)\\ & P(B)\;=\;s_{B}B/(A+s_{B}B+s_{M}M)\\ & P(M)=\;s_{M}M/(A+s_{B}B+s_{M}M) \end{array}$$

Hence $s_{B}$ and $s_{M}$ give the relative selective advantage (>1) or disadvantage (<1) experienced by molecules of allele type *B* and *M* respectively, with type *A* treated as a reference. When $s_{B}=\;s_{M}=1$ (the default) the case without selective differences is recovered.

We therefore capture an effective physical bottleneck (sample size N/2) generating random variability upon inheritance, further variance increase due to random reamplification (both of which contribute to the overall genetic bottleneck; Johnston 2019a), paternal leakage with rate *λ*, mutation rate *μ*, and selection according to a fitness function f(A,B).

Under simulated DUI, the sex of an offspring determines the parent from which it inherits all or most of its oDNA. Male offspring inherit most of its oDNA from their father with leakage from their mother, while female offspring inherit as described above. In the case of DUI with zero leakage, inheritance of oDNA is exclusively from the parent of the same sex. This reflects the germline but not the somatic situation in multicellular eukaryotes employing DUI (see Discussion). Other model structures (deterministic reamplification, deterministic leakage, and heteroplasmic initial conditions) are demonstrated in Fig. S1.

### Environmental Change

We model the environment of a population through the fitness function f(A,B). We will consider two different environments:


[M7] $$\begin{array}{rl} & f_{A}(A,B;\sigma ,\epsilon )=A+\sigma B-\epsilon \;\min(A,B)\\ & f_{B}(A,B;\sigma ,\epsilon )=B+\sigma A-\epsilon \;\min(A,B) \end{array}$$

where σ≤1 is the fitness contribution from the less-favored allele in a given environment, and ϵ≥0 is a heteroplasmy cost, penalizing mixed oDNA populations. *M*, the dysfunctional mutant type, always contributes zero to fitness. Before considering selective differences, we will use a simple case


[M8] $$f_{0}(A,B)=f_{A}(A,B;1,0)=f_{B}(A,B;1,0)$$

where both alleles contribute equally to fitness, and there is no heteroplasmy penalty.

We use two different protocols to consider environmental change. The first (as in Fig. 1b to e) involves a single shift, from the environment favoring *A* to the environment favoring *B*, at a timepoint *τ*:


[M9] $$f_{\tau }(A,B,t;\sigma ,\epsilon )\;=\;\{\begin{array}{c} \;f_{A}(A,B;\sigma ,\epsilon )\;\;\;\mathrm{if}\;t\;<\;\tau ;\\ \;f_{B}(A,B;\sigma ,\epsilon )\;\mathrm{otherwise} \end{array}$$

In these experiments, a population is initialized with identical *N* and *λ*, and the mean fitness of the population tracked over time (and particularly measured at time t=500, long after the environmental switch). Our default values are σ=1/2,ϵ=0.

The other environmental protocol is an oscillating one, which continually switches between the environment favoring *A* and the environment favoring *B*, with period *τ*:


[M10] $$f_{\tau }(A,B,t;\sigma ,\epsilon )\;=\;\{\begin{array}{c} \;f_{A}(A,B;\sigma ,\epsilon )\;\;\;\mathrm{if}\;t\;<\;\tau ;\\ \;f_{B}(A,B;\sigma ,\epsilon )\;\mathrm{otherwise} \end{array}$$

In these experiments, a population is initialized with a range of *N* and *λ* values and allowed to compete over many environmental oscillations. In each case, for genetic initial conditions, we set half the males and half the females to be homoplasmic for *A* and half of each to be homoplasmic for *B*.

Some illustrative trajectories of the single-switch model (over a shorter timescale) are shown in Fig. 1b and c. The reader will notice similarities with, for example, the model of (Roze et al. 2005), but with some additional mechanisms, the inclusion of additional oDNA alleles, and a time-varying fitness function (Radzvilavicius and Johnston 2022).

### Implementation

The model was implemented in custom-written C code. R (R Core Team 2022) was used for visualization, with packages *reshape2* (Wickham 2007) and *dplyr* (Wickham et al. 2023) used for data curation and *ggplot2* (Wickham 2016), *metR* (Campitelli 2021), *viridis* (Garnier et al. 2024), and *ggpubr* (Kassambara 2023) used for visualization.

## Supplementary Material

evag161_Supplementary_Data

## Data Availability

This study did not generate new data. All simulation code is publicly available at https://github.com/StochasticBiology/odna-inheritance.
