# Supplementary material for: Optimal Organelle Inheritance Strategies Under Different Changing Environments and Mutational Pressures
Source: Genome Biol Evol. 2026 Jun 27;18(7):evag161. doi: 10.1093/gbe/evag161 (PMC13351738; doi:10.1093/gbe/evag161)
Supplement: evag161_Supplementary_Data [file evag161_supplementary_data.pdf]

## Supplementary Information

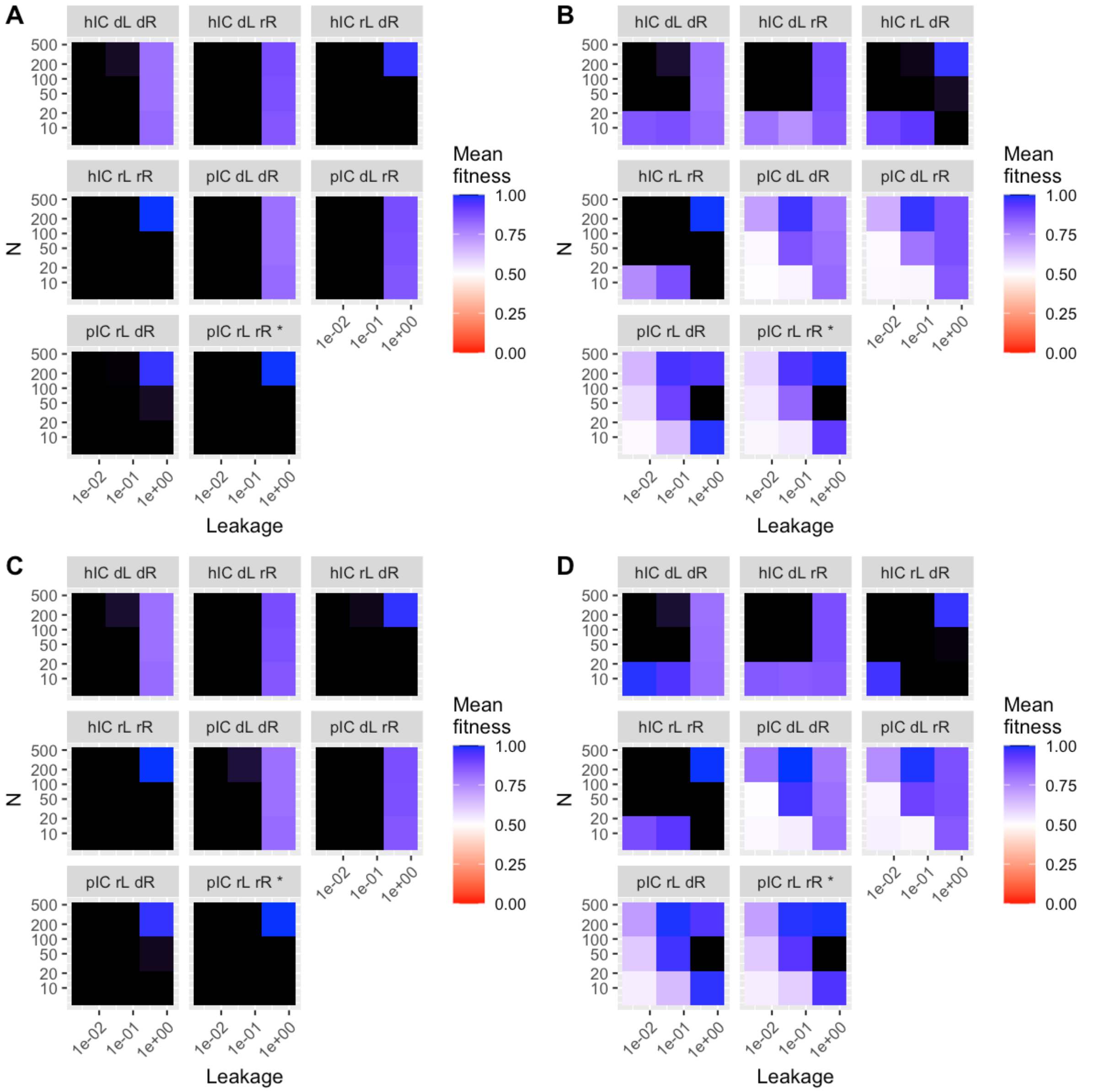


Supplementary Figure 1. **Fitness profiles under different models. (A)** Static environment, no DUI; **(B)** Environmental change period $\tau = 10$, no DUI; **(C)** Static environment, DUI, **(D)** Environmental change period $\tau = 10$, DUI. Panels are labelled by model structure: h/p IC, heteroplasmic/pure (homoplasmic) initial conditions; d/r L, deterministic/random leakage; d/r R, deterministic/random reamplification. Asterisk shows model structure used in the main text. Heteroplasmic initial conditions dramatically change the dynamic profile, as leakage is no longer needed to create heteroplasmy. Deterministic leakage challenges adaptation; deterministic reamplification has a less pronounced effect (mimicking a slightly looser bottleneck). Parameters $N_{pop} = 100$, $\sigma = 0.5$, $\varepsilon = 0$, $s_B = s_M = 1$, $\rho = 0$.

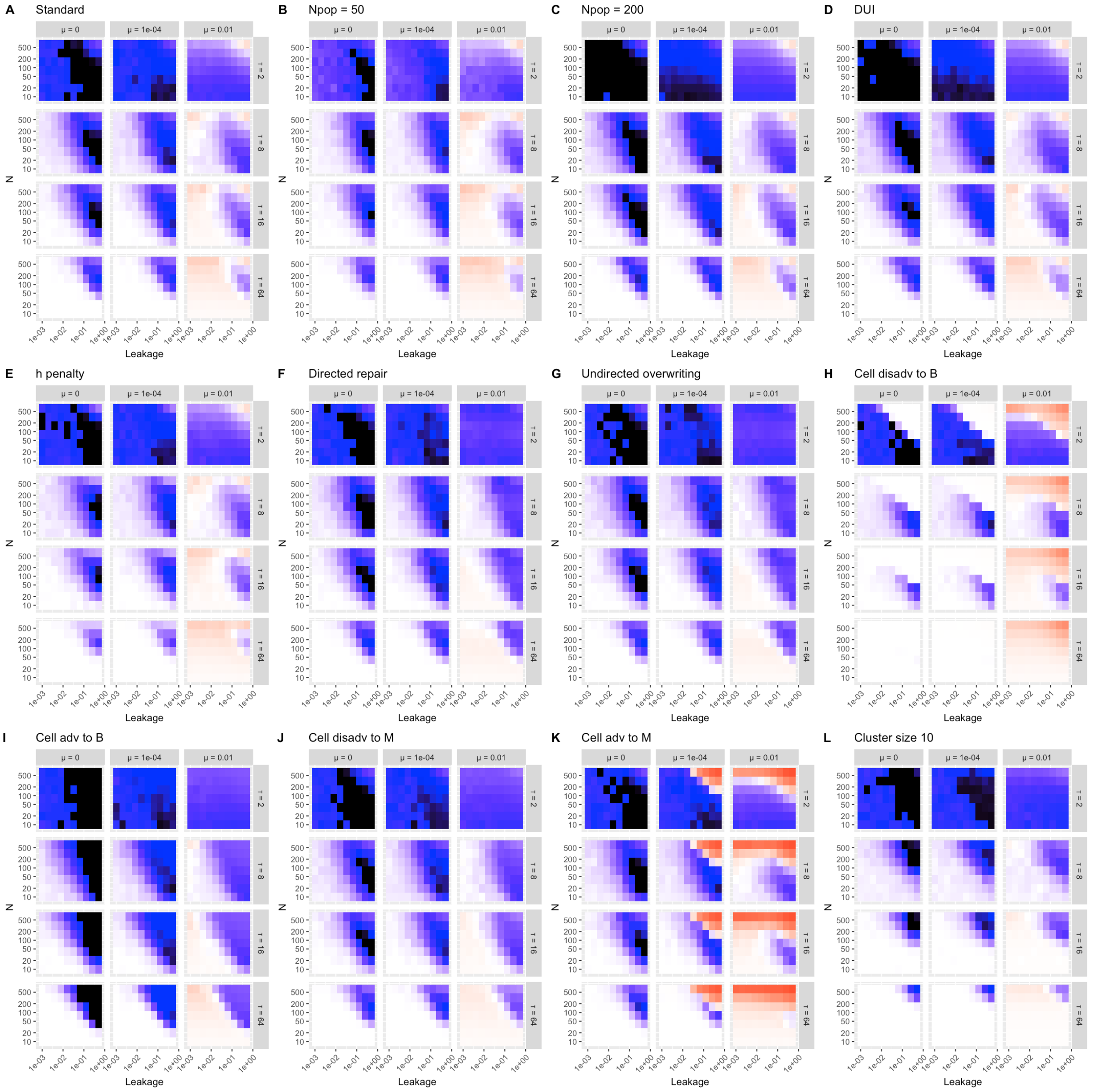


Supplementary Figure 2. **Strategy performance under model variations.** Subsets of fitness plots with different inheritance strategies (horizontal axis, leakage; vertical axis, N) under different external challenges (mutation rate, columns; environmental change period, rows) for different models discussed in the main text and with the same colour scheme (black, highest fitness; blue, high fitness; red, low fitness). **(A)** Default parameterisation ($N_{pop}$ = 100, σ = 0.5, ε = 0, $s_B = s_M = 1$, ρ = 0, as in Fig. 3). **(B)** Smaller ($N_{pop}$ = 50) and **(C)** larger ($N_{pop}$ = 200) organismal population. **(D)** Doubly uniparental inheritance. **(E)** Templated repair with rate constant ρ = $10^{-3}$. **(F)** Heteroplasmy penalty ε = 0.25. **(G)** Doubly uniparental inheritance with heteroplasmy penalty ε = 0.25. **(H)** Within-cell selective disadvantage to B ($f_B$ = 0.9) and **(I)** advantage to B ($s_B$ = 1.1). **(J)** Within-cell selective disadvantage to M ($f_M$ = 0.9) and **(K)** advantage to M ($s_M$ = 1.1). **(L)** Within-cell selective advantage to M ($s_M$ = 1.1) with templated repair at rate constant ρ = $10^{-3}$.

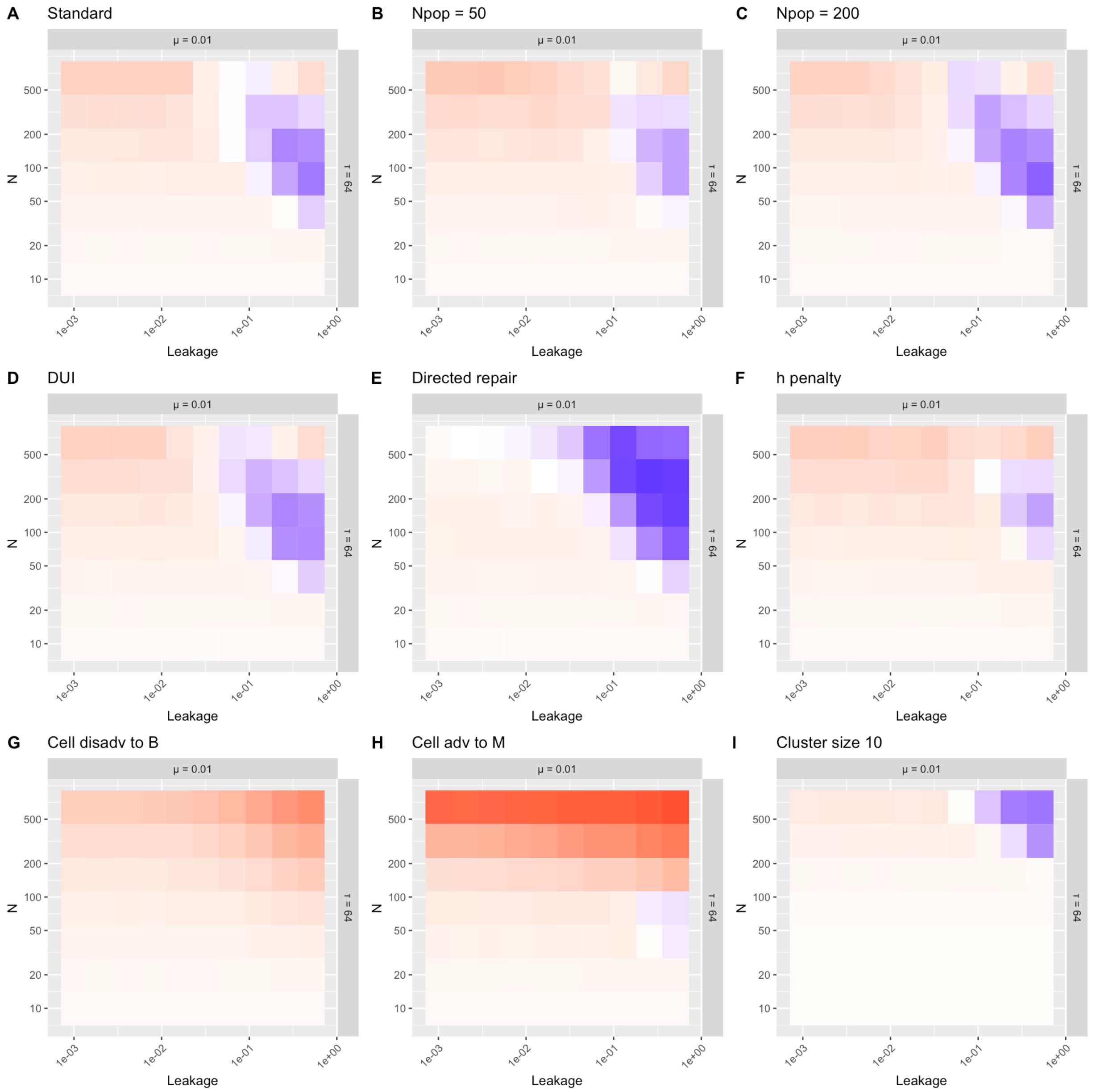


Supplementary Figure 3. **Influence of model variations at high mutation rate.** Analogous plot to Fig. 4, for higher mutation rate 0.01. Mean fitness is plotted as in Fig. 3, with exactly the same colour scale (blue, high fitness; red, low fitness, without Fig. 3's adjustment). **(A)** Default parameterisation ($N_{pop}$ = 100, σ = 0.5, ε = 0, $s_B$ = $s_M$ = 1, ρ = 0, as in Fig. 3). **(B)** Smaller ($N_{pop}$ = 50) and **(C)** larger ($N_{pop}$ = 200) organismal population. **(D)** Doubly uniparental inheritance. **(E)** Templated repair with rate constant ρ = $10^{-3}$. **(F)** Heteroplasmy penalty ε = 0.25. **(G)** Within-cell selective disadvantage to B ($s_B$ = 0.9). **(H)** Within-cell selective advantage to M ($s_M$ = 1.1). **(I)** Inheritance of clusters of size $n_c$ = 10 rather than individual molecules.
